# Development and validation of a Nurse Station Ergonomics Assessment (NSEA) tool

**DOI:** 10.1186/s12912-021-00600-8

**Published:** 2021-05-31

**Authors:** Hamidreza Mokarami, Sahar Eskandari, Rosanna Cousins, Mahmood Salesi, Reza Kazemi, Mohsen Razeghi, Alireza Choobineh

**Affiliations:** 1 Departemt of Ergonomics, School of Health, Shiraz University of Medical Sciences, Shiraz, Iran; 2 Department of Psychology, Liverpool Hope University, Liverpool, UK; 3 Chemical Injuries Research Center, Systems Biology and Poisonings Institute, Baqiyatallah University of Medical Sciences, Tehran, Iran; 4 Department of Physiotherapy, School of Rehabilitation Sciences, Shiraz University of Medical Sciences, Shiraz, Iran; 5 Research Center for Health Sciences, Institute of Health, Shiraz University of Medical Sciences, PO Box 71645-111, Shiraz, Iran

**Keywords:** Nursing workstations, Ergonomic assessment, Psychometric properties, Working environment, Hospitals

## Abstract

**Background:**

Nurse stations are one of the primary units for supporting effective functioning of any hospital. They are important working environments that demand adherence to known ergonomic principles for the well-being of both staff and patients. The aim of this study was to develop a psychometrically tested tool for the assessment of the ergonomic conditions of nurse workstations in hospitals.

**Methods:**

Ten hospitals, with a total of 133 nurse stations participated in this mixed-methods research. The domains and items of the tool were developed based on a literature review, an experts’ panel, and interviews with nurses.

**Results:**

The final nurse station ergonomic assessment (NSEA) tool has good psychometric properties. Validity was assessed by face validity and content validity. Reliability was evaluated using inter-rater agreement and test-retest reliability analyses with a four-week interval between assessments. The NSEA is comprised of 64 items across eight domains: layout and location (7 items), workspace (11 items), security-safety (5 items), environmental conditions (8 items), counter (8 items), chair (13 items), desk (9 items), and monitor (3 items).

**Conclusions:**

The NSEA adds to the literature a tool for managers to ensure they comply with legal requirements and support best practice for those working on hospital wards. The NSEA can be used to identify challenges for healthcare professionals who use nurse stations and support the execution of targeted interventions to improve human-environment interactions.

**Supplementary Information:**

The online version contains supplementary material available at 10.1186/s12912-021-00600-8.

## Background

Good design of hospital buildings is important to support both the healing processes that take place inside them, and the health and safety of those who work in them. Ward design can impact on behaviour [1]. The nurse station is a key area of human-environment interaction in hospital wards. The relationship of physical design, work processes, technology infrastructure and organizational culture in a nurse station underpins nurse job satisfaction and retention, work-related stress and patient safety and care [2]. Patients, staff and all stakeholders benefit from thoughtful planning of hospital spaces that follow proven ergonomic principles. Hendrich and Chow [2] suggested that hospital chief executive officers should ask “Does the physical space reflect evidence-based standards known to enhance caregiver and patient experience?” (p. 16). To facilitate such an analysis of nurse stations, there is a need for a standard analysis tool. The aim of this study was to develop and validate a nurse station ergonomic assessment tool to address this gap in the literature.

Ergonomics is the science of understanding the interaction of people and work systems. It is a multi-disciplinary approach, underpinned by three sets of interrelated factors [3]. Physical factors (anatomical, anthropometric, physiological systems) harness human capability issues relevant to efficient and effective workplace layout and working environment. Cognitive factors focus on mental process pertinent to handling information, interpretation, task analysis, human-machine interface, workload, alarm philosophies, and involve human senses (vision and hearing, touch, taste, smell). Organizational factors (sociotechnical systems, cooperation, participation) are important for managing work responsibilities, work procedures and communication processes. There are established ergonomic principles that can be applied in the design of nurse stations to encourage good performance, and ameliorate the high levels of musculoskeletal disorders and cumulative trauma disorders associated with nursing [4, 5].

The nurse station is typically a hive of activity, and one of the key sections of any hospital. It is the place where nurses work and communicate with other nurses, physicians and administrators, and with patients, their families and other visitors. Nurse stations should provide a functional space for coordinating patient care responsibilities, communication, and documenting patient records [6]. In-patient healthcare requires good teamwork, and the location, arrangement, accessibility, visibility, furnishings, workspace design and seating arrangements in nurse stations play a significant part in supporting this work [7].

**Fig. 1:**
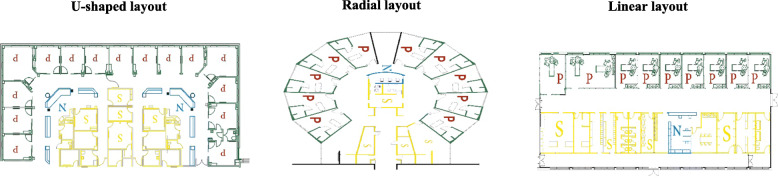
Common examples of centralized nurse station and ward layout. (P represents patients’ rooms, N the nurse station, and S service and support spaces)

The best layout and location of a nurse station requires an understanding of the ward functions, work zones, floor plan, and the communication and chart management systems. Layout and location are important to maximize care time and minimize travelling time. It is estimated that nurses spend about one-third of their time walking in the ward [8]. Visibility of patients from work areas is significant in improving safety outcomes [9]. The position of nurse stations with respect to the patient rooms is discussed in terms of being centralized, or decentralized [10]. Traditionally, one centralized nurse station has been the primary work area of a ward. The introduction of electronic medical records provided the option of using mobile computers and having several subunits or decentralized nurse stations. There is inconsistency in studies that have examined the benefits according to location of nurse station. Centralized workstations have different layouts to provide best oversight of most of the patients in the ward (See Fig. 1). Decentralized nurse stations usually provide good visibility because of being located between two patient rooms [11]. Durham and Kenyon [12] concluded that decentralized nurse stations can provide increased patient care and satisfaction, that walking distances for nurses did not differ between the two types of nurse station, and there were disadvantages for decentralized nurse stations in terms of reduced perceptions of teamwork, reduced communications with peers, and increased feelings of isolation. In summary, we concur with the conclusions that there are costs and benefits to both types of location [10].

Nurse stations must be large enough to accommodate multiple workers [13], and have enough appropriate space and dimensions for carrying out the diverse activities undertaken by staff [14]. There is a need for deep counters for working on traditional paper charts, and sufficient space around computers to open and use patients’ paper files remains, even though there has been a move to storing most documentation electronically. The literature review of Seelye is dated in terms of technology overtaking some of the paperwork involved in healthcare at the time [15]. Nevertheless, Seelye’s point that it is possible to efficiently design a nurse station in which all the required resources, facilities and services are gathered to minimize nurses’ walking time, and support maximum contact opportunities with patients, has, we suggest, stood the test of time.

All aspects of chair design should be easily adjustable, to account for the different anthropometrics of all users of a nurse station. Musculoskeletal disorders resulting from the use of ill-fitting furniture can lead to the prolonged absence of the staff [16], and is associated with increased nursing errors [17]. Proper lighting at nurse stations reduces eye tension and improves visual conditions greatly [18]. Minimizing glare from bright sunlight, reflection on screens and shiny surfaces is also relevant [19].

Noise is difficult to control in busy hospitals, but there is a need for noise control that revolves around the work. Nurse station design should incorporate areas that give speech privacy whether in person or on the telephone. Best practice would dictate a dedicated space for confidential conversations [20]. Undesirable noise impairs human performance [21], and is a significant predictor of distress [22], burnout and increased the likelihood of errors among nurses [23].

Ventilations systems are also an important consideration for the nurse station and wards, if the best type to use may differ. For example, natural ventilation is suitable for warm and temperature climates, and even opening a window can improve infection control in areas of the world which have strong winds and limited capital [24]. Mechanical ventilation has the benefit of being controllable, if more expensive to install and run. Hybrid systems in which natural ventilation is the default and mechanical ventilation is reserved for when natural driving forces are too low are ecologically beneficial. Maximising natural ventilation strategies in hospital wards does not need to compromise thermal comfort [25].

The evidence we have summarized here indicates that the ergonomic status of nurse stations can be readily assessed using a standard and valid tool. Such an assessment could be used as the basis for designing and implementing targeted ergonomic interventions. To the best of our knowledge, however, there has not yet been published an easy to use tool for this purpose. Therefore, the aim of this study was to develop and validate a tool for the assessment of the ergonomic conditions of nurse stations.

## Method

### Research design

**Fig. 2:**
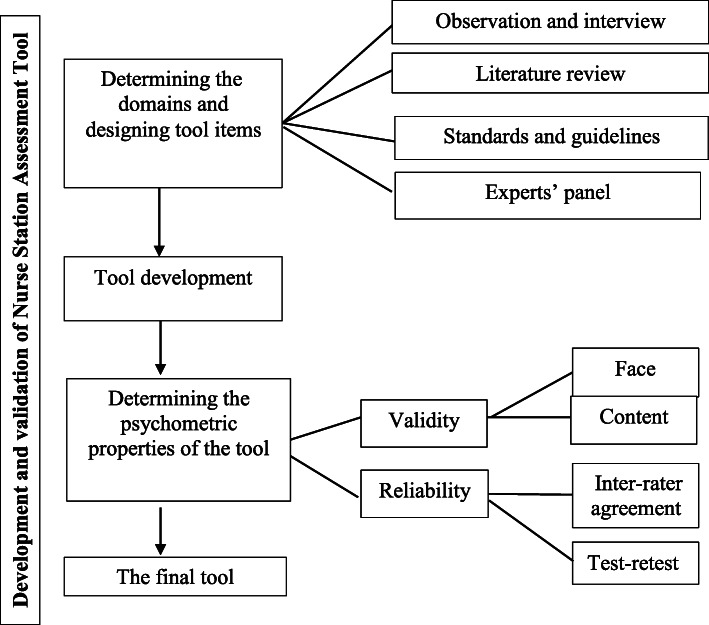
Mixed methods sequential explanatory study design

A mixed methods sequential exploratory design was used to develop a tool for profiling the ergonomic conditions of nurse stations (see Fig. 2). The qualitative part of the study sought to identify the components and scope of work associated with nurse stations using the researchers’ observations and the ideas of a panel of experts. These were considered alongside unstructured interviews with nursing staff, a literature review, and the guidance in *Standards for Planning and Design of Safe Hospitals* [26]. The quantitative part of the study was an evaluation of the psychometric properties of the tool and associated revisions. The study was conducted in ten educational hospitals affiliated to Shiraz University of Medical Sciences during a period of 12 months. All participants provided written informed consent.

### Tool item design and dimensions

#### Literature review

A review of the literature was undertaken by two members of the research team following standardized methodology. First, Scopus, PubMed, Web of Science, and Embase databases were searched to find the important domains and factors in the design of nurse stations in published articles. Inclusion criteria were access to the detailed content, and the paper addressed various factors in the design of nurse stations in English. The primary search terms were “Nurse Station”, “Nurse Unit”, “Nursing Ward”, “Centralized Design Nurse Station”, “Decentralized Design Nurse Station”, “Hybrid Nurse Station”, “Patient Care Unit Design”. Secondary BOOLEAN searches incorporated “Ergonomics” and “Safety”. No date constraints were put on the search. In addition, we included a search of ergonomics and human factors textbooks and research papers published by healthcare furniture manufacturers for aspects and recommendations on nurse station design. After searching for articles and preparing an initial list, the titles were studied, and repetitive cases were omitted. Lastly, the full texts of the remaining articles were analysed, and relevant domains and factors were extracted.

#### Standards for planning and Design of Safe Hospitals

Iran’s Ministry of Health, Treatment, and Medical Education has developed *Standards for Planning and Designing Safe Hospitals* [26]. To date, this comprises 15 volumes of comprehensive regulations and guidance that cover all aspects of hospital design. This publication was scrutinized by two members of the research team and the important factors for designing the layout of nurse stations were extracted. The *Standards for Planning and Designing Safe Hospitals* was commissioned to update the physical planning and design of Iranian hospitals to accommodate the considerable developments in medical, technical and electronic processes since the previous guidelines 70 years earlier. Whilst the regulations and associated guidance to implement the Standards are country-specific, they were derived from almost 5000 national and international documents. As they are based on the same evidence, the Iranian Standards are very similar to various other international and national Standards. These include the *International Health Facility Guidelines* [27], the United Kingdom government’s *DH health building notes* [28], and *Guidelines for Design and Construction of Hospitals and Healthcare Facilities* [29] which originated in the United States.

#### Field observation and interviews

Five of the ten hospitals associated with the study were randomly selected, and the ergonomic characteristics of the nurse stations were assessed by the research team using the guidance in *Standards for Planning and Designing Safe Hospitals* [26]. Simultaneously with the field observations, unstructured interviews were conducted with 34 nurses who were based at a sample of the nurse stations in these hospitals. The interview guide (see Additional File 1) was newly developed for this study. It followed the normal procedure in grounded qualitative research where the interviewer first asked an open-ended question, then the interviewee’s primary answer was followed by further spontaneous probing questions based on their reflection of that answer. The goal was a full understanding nurses’ perception of good nurse station design based on their tangible work experiences. The interviewer had both training and experience in this form of data collection.

Thus, in the interviews, all the nurses were first asked a single general question: “What problems and concerns do you have regarding your workstation?” According to the interviewees’ responses, they were asked to elaborate on the issues they raised, and their responses were recorded. Finally, the transcribed data were analysed based on the content analysis method [30], and the important factors in the design of nurse stations were identified from the perspective of nurses. These are collated in Table 1.

**Table 1:** Nurse Station Work Design Features by Priority (*N* = 34)

Nurses’ Important Work Design Features	Nurses’ ratings Frequency / Percent
Nurse stations should be located at the centre of the ward, and not next to the entrance	32 / 94%
There should be enough space for nurses’ and physicians’ equipment and tools	30 / 88%
Nurse stations should allow direct observation of all patients	29 / 85%
Nurse stations must have at least two entrances, each with doors to prevent unauthorized access	27 / 79%
Nurse stations should have dedicated space for meetings	23 / 68%
The layout of desks and equipment such as emergency trolley and nurse call systems should permit easy access	20 / 59%
The ward entrance and exit should be visible from the nurse station	19 / 56%
Nurse stations should have fully adjustable chairs with strong legs	19 / 56%
Nurse stations should have clear access to the drug store	16 / 47%
Nurse stations should have good ventilation	14 / 41%
Nurses form and size should not be prescribed, but bespoke, according to the layout of beds	13 / 38%
The counter should have adjustable heights to allow for standing and sitting	13 / 38%
A chart processing area should be located in a quiet part of the nurse station	12 / 35%
The nurse station counter must have a toughened glass screen for nurses’ safety	12 / 35%
The counter depth should all medical records to be opened up	12 / 35%
There must be a handwash basin	8 / 24%
The nurse station counter area should have drawers for storage	8 / 24%
Nurse stations should have a charting desk	8 / 24%
The floor finish should prevent slipping	8 / 24%
Nurse stations should have a window	7 / 21%
Work surfaces should not have sharp edges	7 / 21%
All wires and cables in a nurse station should be placed in a duct	6 / 18%
There should be a sufficient number of computer desks with space for monitor and keyboard for the number of nurses working	5 / 15%
Nurse stations should have variable lighting according to the work	5 / 15%
Colours in addition to white should be used	5 / 15%
Shelves for forms and files should be designed under the counter	4 / 12%
The trolley of patient files should be next to the charting desk	4 / 12%
Flowers and plants should be used around the nurse station	2 / 6%

The process of encoding and extracting the domains was performed separately by two members of the research team. The agreement of themes was confirmed by using Holsti’s formula (Reliability = 2 m/N_1_ + N_2_, where m is number of coding decisions where coders agree, and N_1_ and N_2_ are number of decisions made by the coders) [31]. Reliability was good (> 80%). Disagreements were resolved by considering associated field notes.

#### Experts’ panel

The findings from the previous three stages were discussed and revised by a panel of ten experts working in ergonomics, occupational health, and nursing during three sessions. After reaching a general agreement on the items, and their allocation into eight distinct dimensions, operational definitions were established according to standard international conceptualizations and definitions in the literature (e.g. [4, 13, 32]) (see Table 2). A checkback confirmed all items were appropriately assigned in one of the domains.

**Table 2:** Operational definitions of eight domains of ergonomic assessment of nurse stations

Domain	Operational Definition
Layout and location	The floorplan of the nurse station, and component parts, in relation to the hospital ward it serves.
Workspace	The dedicated physical place where health professionals and administrators spend a significant proportion of their time. Activities include monitoring and responding to patient status, providing therapeutic patient care, documenting interventions, supporting referrals, admissions, tests required, transfers, and release of patients.
Safety-security	Physical structures to support the physical safety of healthcare staff
Environmental conditions	Sensory input that can support or interfere with patient care. These include lighting type, levels, and controls; noise type, levels and controls; glare-producing surfaces; slippery surfaces; ventilation; air-conditioning and heating levels and controls.
Counter	Furniture that serves to receive healthcare professionals, patients and visitors. A workspace for sharing information using documents and equipment with users of a hospital ward. Can include a surface, lights, and under counter space. May be open, and /or include lockable screen and shutters.
Desk	Furniture providing individual physical workspace. May be used seated or standing.
Chair	Furniture providing individual seating for work in nurse station.
Monitor	Computer screen.

### The psychometric properties of the tool

After the development of a provisional version of the tool, the process of determining the psychometric properties was conducted as follows. The conventional alpha level of *p* < .05 was used to determine statistical significance.

#### Face and content validity

The provisional tool was sent to ten professors of occupational health, nursing, and ergonomics. They were asked to check the grammar, wording, and item allocation for each item. Where they perceived any non-compliance with these principles, they should provide a suggestion for improving the item. In addition, a survey was conducted with 15 nurses in order to resolve any ambiguity and understandability of the items for them. The comments of the professors and the nurses were collated and discussed among the research team members, and the necessary changes were made.

To determine the validity of the revised tool, it was evaluated in terms of Content Validity Index (CVI), Content Validity Ratio (CVR), and Impact Score. In doing so, two separate files were sent to 15 university professors and experts in the subject of study. The first file was to examine CVI: the experts were asked to examine the three criteria of relevance, clarity, and simplicity for each item separately [33]. Subject evaluation of items was in accordance with Polit et al.’s recommendations that items with CVIs of more than 0.79 are acceptable, those between 0.7 and 0.79 needed to be reviewed, and those items with a CVI less than 0.7 were unacceptable and should be removed. Ultimately, a valid assessment tool would comprise items yielding a minimum average CVI of .80 [33]. The second file was to examine the degree of necessity for each item to calculate CVR [34]. According to the table Lawshe designed, which gives figures based on the number of experts participating in the evaluation, items with CVRs > 0.49 (for 15 experts) were important (significant, *p* < 0.05), and those with lower CVRs had to be removed [34]. Finally, item impact scores were examined. Ten nurses were asked to review and score each of the items in terms of their importance using a 5-point Likert Scale (1 = not important, 5 = very important). Items with an impact score greater than 1.5 were retained [35].

#### Reliability

The reliability of the tool was evaluated using inter-rater agreement coefficient. Nine nurse stations in one of the hospitals were evaluated by six ergonomics experts. After four weeks, the same nine stations were re-evaluated. To investigate the agreement coefficient between the experts, the Intra-class Correlation Coefficient (ICC) was used with a confidence level of 95%. Spearman’s correlation coefficient was also used to examine the correlation between the total scores of the tool in the first and the second stages of evaluation with a four-week interval. Additionally, all the nursing workstations of the ten hospitals studied (*n* = 133) were evaluated by two experts separately. To investigate the agreement coefficient between two experts for all items of the tool, Cohen’s Kappa coefficient was used.

## Results

### Tool item design and dimensions

#### Literature and standards review

The literature review showed that the most important ergonomic factors in the design of the nursing station were the location of nurse stations, observation of patients, access, spatial layout, walking distance, thermal comfort, sound level, adequate lighting, storage space, daylight, ventilation, ergonomic furniture, routing, hand hygiene facilities, construction materials, safety, and security. This has been outlined and discussed in the Introduction, as well as contributing to the design of the tool.

Key findings to support decision making on the adequacy of ergonomic factors for guiding assessment were that according to Feiler and Stichler [13], nurse station counters should be at least 150 cm long and 60 cm deep to accommodate monitoring and reporting equipment, although medical furniture manuals typically indicated 90–120 cm per person (e.g. [36]). There was general agreement with Feiler and Stichler’s specification that workstations should be 85–90 cm high for leg room when sitting and adjustable according to height to allow work when standing [13]. There were also clear direction of lighting levels, such that a luminance ratio of 500 lx (monitor working area): 300 lx (surrounding work area): 100–200 lx (external area) is recommended for good vision [37].

Woo et al. [38] provided international ergonomic standards for seating at computer workstations. They reported that chairs should have a back rest, the seat height should be adjustable between 38 cm and 56 cm, seat depth should be adjustable between 38 cm and 56 cm, and seat width at least 45 cm. The seat covering should be fabric to minimize static, to provide sliding resistance, and to resist perspiration. Seat coverings should also be easy to clean. Recommendations for best viewing distance from monitors vary substantially from 35 to 85 cm, however Woo et al. suggested that changing font size is a better answer than moving monitors to suit viewers visual capabilities [38].

Study of the “nurse station” sections in the *Standards* [26] showed that factors including patient monitoring, accessibility, nurses station location, charting space, counter dimensions, lighting, secretarial place, and storage, were important in designing an effective nurses’ station [29].

#### Field observations and interviews

Field observations from the nurse stations showed many physical, cognitive and organizational ergonomic problems that were pertinent for the development of an assessment tool, and associated guidance. Important challenges were insufficient workspace, inappropriate location in the ward, inadequate space for computer terminals and keyboards, inappropriate layout of tools, furniture, and equipment, inappropriate height, inadequate depth of the counter, lack of foot space under the counter, non-ergonomic chairs, lack of standing workstations, insufficient lighting, slippery areas, difficult access, and disorganization at the workstations. Similarly, the interviews with nurses showed that they were faced with various problems at their workstations. Their remarks pointed out issues related to layout, workspace, furniture, and lighting. The most important principles for designing nurse stations based on the ideas of the nursing staff who work at them are provided in Table 1.

#### Experts’ panel

According to the results of the previous three stages and the experts’ panel discussions, eight domains were developed for the tool: layout and location, workspace, safety-security, environmental conditions, counter, desk, chair, and monitor. Then, based on the operational definitions for these domains (see Table 2), 92 items were designed.

### The psychometric properties of the tool

#### Content validity

Based on the findings of CVI and CVR analyses, 28 of the initial 92 items were identified to be inappropriate. Therefore, the number of items was reduced to 64. The mean CVI and CVR of the 64 items were calculated as 0.88 and 0.70 respectively, indicating appropriate content validity from the experts’ viewpoints. The results also showed that the impact scores of all 64 items were higher than the minimum acceptable value (> 1.5); the mean score was 4.1.

#### Reliability

The results showed excellent agreement among the experts. Accordingly, the ICC was higher than 0.9 in all eight domains of the tool. The ICC (total mean score) was calculated to be 0.98 (*p* < 0.001) at the first stage and 0.97 (*p* < 0.001) at the second stage. Spearman’s correlation coefficient between the mean scores of the tool in the first and second stages was equal to 0.92 (*p* < 0.001) (see Table 3). This test-retest coefficient indicated very good reliability. Moreover, the mean Cohen’s Kappa Coefficient between two experts was 0.94 for the evaluation of all nursing workstations (*N* = 133).

**Table 3:** Correlation coefficients for domains of the Nurse Station Ergonomic Assessment Tool

Domain	ICC (CI95%) First stage	ICC (CI95%) Second stage	Spearman’s *r*
Layout and Location	0.995 (0.986–0.999)	0.996 (0.989–0.999)	0.984^**^
Workspace	0.951 (0.876–0.987)	0.915 (0.786–0.978)	0.917^**^
Safety-security	0.971 (0.925–0.992)	0.949 (0.871–0.987)	0.807^**^
Environmental conditions	0.954 (0.833–0.988)	0.975 (0.936–0.993)	0.884^**^
Counter	0.942 (0.853–0.985)	0.922 (0.802–0.980)	0.878^**^
Chair	0.975 (0.937–0.933)	0.932 (0.828–0.982)	0.998^**^
Desk	0.995 (0.987–0.999)	0.996 (0.990–0.999)	0.922^**^
Monitor	0.986 (0.966–0.996)	0.962 (0.903–0.990)	0.848^**^
Total	0.984 (0.961–0.996)	0.975 (0.936–0.993)	0.918^**^

** *p* < .001. ICC - Intra-class correlation coefficients. CI95–95% Confidence Interval

The final nurse station ergonomic assessment (NSEA) tool included eight domains and 64 items as follows: layout and location (7 items), workspace (11 items), security-safety (5 items), environmental conditions (8 items), counter (8 items), chair (13 items), desk (9 items), and monitor (3 items). The items included in the NSEA are presented in Table 4.

**Table 4:** The Nurse Station Ergonomic Assessment Tool

Ward profile:	Name of hospital:			
Domain	Item	Yes	No	Comments
**Layout and location**	1. The location of the nurse station is in accordance with the design of the section and in the centre of the patients’ rooms			
	2. The nurse station is not in the way of ward traffic			
	3. The nurse station is in a location where the entrance is visible			
	4. All patients can be directly observed and monitored from the nurse station			
	5. The nurse station is located to allow broadly equal access to all patients			
	6. The nurse station is located to allow good communication and easy access and view of the medication room			
	7. The nurse station is located to allow good communication and easy access to storage space for required medical equipment			
**Workspace**	8. The charting space is embedded in the quiet part of the nurse station			
	9. There is adequate space for a charting system in the nurse station			
	10. The nurse station includes a separate space for secretarial activities			
	11. A separate space for group meetings is provided in or near the nurse station.			
	12. The nurse station includes a permanent space next to the charting desk for the medical records trolley			
	13. At the nurse station, there are drawers and shelves for keeping files, records, and medical forms			
	14. The height of the shelves and cabinets at the nurses’ station is easily accessible to nurses			
	15. In the nurse station, the placement of equipment such as cabinets, desks, monitors, nurse call system, etc. is appropriate			
	16. At the nurse station, equipment, items and fixtures that are used frequently are readily available			
	17. The dimensions of the nurse station are proportional to the space, facilities, equipment, and the number of nurses and physicians per shift			
	18. The nurse station allows a choice of working sitting or standing			
**Safety-Security**	19. At critical times, staff can easily enter and exit the nurse station			
	20. Security measures are in place to prevent non-authorized people from entering the nurse station			
	21. The nurse station includes facilities to maintain the health and safety of nurses			
	22. A duct is used to cover all wires and cables in the nurse station			
	23. The furniture (shelves, counters, etc.) in the nurse station are securely fixed and suitable for the load they support			
**Environmental Conditions**	24. There is correct lighting in the nurse station to perform tasks			
	25. Lighting is uniformly distributed at all points in the nurse station			
	26. An appropriate combination of yellow and white lights is used at the nurse station			
	27. The nurse station has a window to provide natural light			
	28. There is an acceptable sound level in the nurse station and its surrounding areas			
	29. The temperature of the nurse station is adjustable and maintained at a comfort level			
	30. There is an air conditioner system in the nurse station			
	31. The air conditioner system is effective			
**Counter**	32. The design of the counter enables patients using a wheelchair to see and communicate with nurses			
	33. The counter surface dimension is sufficient for writing activities			
	34. The dimensions of the counter surface level are adequate for placing computer equipment and other necessary accessories			
	35. The counter surface edges are not sharp			
	36. The counter surface level is not rough			
	37. The light reflection over the surface is not bothering			
	38. Under the counter surface, there is enough space for nurses to move their feet			
	39. Nurses can rest their feet can rest on the floor or another support when sitting behind the counter			
**Chair**	40. The seat height of chairs in the nurse station is easily adjustable			
	41. The chairs in the nurse station have armrests			
	42. The height of armrests can be adjusted			
	43. The dimensions of the armrests of the chairs in the nurse station provide good support for nurses’ forearms			
	44. The armrests of chairs in nurse station do not prevent the worker from approaching the work surface (desk, counter, etc.)			
	45. The chairs in the nurse station support the lower back			
	46. The backrest of chairs in nurse station support the upper extremities			
	47. The seat has an adjustable width and depth, to suit the nurses’ anthropometric features			
	48. The frontal edge of the seat is not sharp			
	49. The seat cover is anti-perspiration and prevents nurses from slipping forward			
	50. The chairs of nurse station have strong legs			
	51. There are swivel chairs in the nurse station			
	52. There are enough chairs at the nurse station			
**Desk**	53. There is a charting desk at the nurse station			
	54. The design of the charting desk provides workspace for several nurses			
	55. The height of desks at nurse station (computer desk/charting desk) is appropriate for the forearm height in sitting position			
	56. The dimensions of the computer desk at the nurse station are suitable for placing the monitor, keyboard, mouse, etc.			
	57. The edges of desks at the nurse station are not sharp			
	58. The surfaces of desks (computer desk, charting desk) are not bothering			
	59. Light reflection from the desk surface (computer desk, charting desk) is not bothering			
	60. Under the surface of desks at nurse station (computer desk, charting desk), there is enough space for nurses to place and move their feet			
	61. While sitting behind desks at nurse station (computer desk, charting desk), the nurses’ feet are supported by the ground or a footrest			
**Monitor**	62. The computer monitor can be placed in an appropriate distance from the nurse			
	63. The monitor is directly in front of the user			
	64. To prevent light reflection, the monitor is perpendicular to the window or light sources vertically			

## Discussion

In this research, an easy-to-use tool for the assessment of the ergonomic conditions of nurse stations was developed for the first time. The results of this study confirmed that the new *NSEA* tool has good psychometric properties. At present, standard guidelines are to be found piecemeal in the academic literature, and lengthy legal documents. The NSEA supports compliance with ergonomic standards for hospital nurse stations that is not currently available. The NSEA is based on international standards of best ergonomic design and should afford informed decisions to be made about nurse workstation design that will improve working conditions and ultimately patient care. It has broad application, and its use should not be confined by ward, hospital type, or geographic region.

We have provided a quick and simple method to identify problems, and support improvements to the ergonomics of nurse stations. The items in the tool are evidenced based and emerged from our mixed methods sequential exploratory design research. This provided eight domains and 64 items. The target for any nurse workstation would be that all 64 items are endorsed “yes”. This would ensure compliance with ergonomic standards, and support workplace health. In practice, there may be some nurse stations that achieve a ‘total score’ less than 64, and there may be differences in the ergonomic standards of workstations even in the same hospital. The level and profile of the NSEA score could be used as a quality improvement tool. Regular assessment will pick up on requirements for maintenance and promote continuous improvement. Ultimately this approach will allow the NSEA to identify challenges for all healthcare professionals who use a particular nurse stations and support the execution of targeted interventions to support effective human-environment interactions.

Issues relating to the domain *layout and location* have been repeatedly emphasized in the literature on nurse stations and remain a consideration for healthcare designers and managers. Ultimately, items in this section of the tool enable managers to consider key functions of the layout, such as the ability to view patients from the nurse station, which has long been a design criterion for specialized care units as well as other nursing units [39]. There are recommendations for the nurse stations in the literature that can be given in guidance, such as those referring to the height of barriers in a ward being under 3 ft high to maintain visibility of patients. This advice remains, but it is not likely to be feasible for a large unit requiring structural columns and walls, or curtains for privacy. Similarly, some equipment may be too tall or bulky to see around. These potentials should be acknowledged, and ways to attain visibility for such hidden areas considered. Perhaps the storage areas most adjacent to the nurse station and entrance could have half-height walls, still allowing views, and electrical / data outlets with enclosed spaces further away from the main desk.

The domain *workspace* relates to various problems that were raised in both field observations and nurse interviews. In spite of the advancement of technology and replacement of paper records with electronic health records, many hospitals still use traditional paper records, which require space at nurse stations. Sufficient space is an important challenge in the physical design decisions in care units but poorly defined, even in legal standards [40]. Assessments should use local knowledge to ascertain the sufficiency of space. Where there is space shortage, then a reconfiguration of furniture may be considered, alongside consideration of whether some documentation could be electronic, and some paper files archived. Some storage areas will need walls or a door for auditory privacy and to avoid visual distractions, such as in the medication room. The development of larger flatter computer monitors can also serve to save space if mounted on a wall for better visibility and to avoid using counter space.

Regarding *Counters, Desks, Chairs* and *Monitors*, one of the basic principles of ergonomics is to pay attention to the furniture proportional to the size differences of the staff [41]. Hotdesking – the same desk and chair being used by more than one person according to availability – requires staff to be able to easily adjust their workstations according to their needs [42]. If the furniture is not comfortable and user-friendly, it will have a negative effect on working style and performance [43], and has been linked to prolonged absence of the nursing staff with multiple skeletal disorders [16]. An assessment for suitability of furniture can be made simply with this ergonomic assessment tool.

The NSEA also considers environmental conditions of the nurse station, such as light, sound, and atmosphere – all important factors that predict staff well-being and occupational performance. These should be regularly reviewed as proper lighting design can improve nursing care and minimize human errors and, as a result, improve the quality of life in therapeutic settings [44]. Noise level is a significant predictor of patient wellbeing, nurse distress and increased likelihood of errors among nurses [23]. Ventilation and thermal comfort can be assessed according to geography and type of ward.

Colour has also been recognized as an important element of design in health centres, and our interviews with nurses indicated that they would favour more colour in their work setting. Colour preference is a cognitive factor, and although there is not enough scientific evidence on the relationship between a specific colour and a particular feeling, some studies have suggested a close relationship between colour perception and individuals’ mental or emotional attitudes [45]. A nurse station is the heart of ward activities, and it is helpful to make it visually bold with appropriate colour and light for the maximum efficiency of the staff [46]. This viewpoint was not directly supported in this research, however there were recommendations from nurses and other sources that colours in addition to white would be beneficial to the workstation.

*Safety-Security* was also included in the ergonomic tool. The safety of medical staff working in nurse stations is also important. A well-designed and safe environment reduces the number of injuries to nurses. Another aspect of safety is the issue of violence against nurses. Fear of violence affects employees’ performance and reduces their response to care needs, especially in emergency situations [47]. This issue was identified as an important factor for the present study tool, as it was exerting high stress to the nurses under investigation. Designing nurse stations that are a secure work environment is therefore essential.

### Limitations

All nurse stations in the hospitals which participated in this study were in a centralized layout, and the nurses had no experience of working in decentralized stations. As such, certain conditions, might have not been taken into account. However, a literature review, reference to standard guidelines for hospital design, and expert opinions were also used for identifying the important factors in ergonomic designing of nurse stations in this research.

The tool we present for assessing the ergonomic configuration of a nurse station makes reference to best practice in the majority of these hospital workplaces. There may be a need for local add-on items for some specialist nursing units.

We did not currently have sufficient data to undertake exploratory factor analysis to provide a full psychometric test of the properties of all items and domains of the *Nurse Station Ergonomic Assessment Tool*. Future developments for the tool include collecting sufficient data for this purpose to confirm the factor structure.

## Conclusion

The NSEA adds to the literature a quick and simple tool for managers to ensure compliance with legal requirements and promote best practice in workplace design standards in hospitals. The tool has good psychometric properties and can be used to identify challenges to those working on hospital wards and support the execution of targeted interventions to improve human-environment interactions.

## Supplementary Information

**Additional file 1.**

## Data Availability

The datasets used and/or analysed during the current study are available from the corresponding author on reasonable request.

## Abbreviations

CVI: Content Validity Index
CVR: Content Validity Ratio
ICC: Intra-class Correlation Coefficient
NSEA: Nurse station ergonomic assessment
