# Supplementary material for: Development and validation of a Nurse Station Ergonomics Assessment (NSEA) tool
Source: BMC Nurs. 2021 May 31;20:83. doi: 10.1186/s12912-021-00600-8 (PMC8165804; doi:10.1186/s12912-021-00600-8)
Supplement: Supplementary file 1 — Additional file 1. [file 12912_2021_600_MOESM1_ESM.docx]

**Additional File 1. Interview Guide**

**Introduction:**

Thank you for agreeing to participate in this study.

We are conducting research to develop and validate a tool for the assessment of the ergonomic conditions of nurse stations. The aim of this interview is to understand what is important to you in terms of your working conditions in the nurse station. My questions will focus on understanding your perception of good nurse station design based on your tangible work experiences. You will be asked to answer in as much detail as possible.

I remind you that participation in this study is voluntary. You may change your mind at any time and discontinue your participation in this study.

Keep in mind the interview will be audio-recorded. No identifying information will be collected, and your voice recording will be deleted after transcription.

Do you have any questions about the study?

**Primary question**

**What problems and concerns regarding your workstation?**

*Explore, as necessary, around*

**1. Layout and location of the nurse station**

**2. Workspace of the nurse station**

**3. Environmental conditions of the nurse station**

**4. Equipment used in the nurse station**

**5. Other problems related to the design of the nurse station**

**Concluding questions**

What else do you think I should be asking you?

Do you want to add anything else to our discussion?

If, when I review my notes I think of something I should have asked you or need clarification on, do you mind if I contact you? How should I contact you?

***Thank you for your time***
